# Mapsembler, targeted and micro assembly of large NGS datasets on a desktop computer

**DOI:** 10.1186/1471-2105-13-48

**Published:** 2012-03-23

**Authors:** Pierre Peterlongo, Rayan Chikhi

**Affiliations:** 1 INRIA Rennes - Bretagne Atlantique, EPI Symbiose, Rennes, France; 2 ENS Cachan/IRISA, EPI Symbiose, Rennes, France

## Abstract

**Background:**

The analysis of next-generation sequencing data from large genomes is a timely research topic. Sequencers are producing billions of short sequence fragments from newly sequenced organisms. Computational methods for reconstructing whole genomes/transcriptomes (*de novo* assemblers) are typically employed to process such data. However, these methods require large memory resources and computation time. Many basic biological questions could be answered targeting specific information in the reads, thus avoiding complete assembly.

**Results:**

We present Mapsembler, an iterative micro and targeted assembler which processes large datasets of reads on commodity hardware. Mapsembler checks for the presence of given regions of interest that can be constructed from reads and builds a short assembly around it, either as a plain sequence or as a graph, showing contextual structure. We introduce new algorithms to retrieve approximate occurrences of a sequence from reads and construct an extension graph. Among other results presented in this paper, Mapsembler enabled to retrieve previously described human breast cancer candidate fusion genes, and to detect new ones not previously known.

**Conclusions:**

Mapsembler is the first software that enables *de novo* discovery around a region of interest of repeats, SNPs, exon skipping, gene fusion, as well as other structural events, directly from raw sequencing reads. As indexing is localized, the memory footprint of Mapsembler is negligible. Mapsembler is released under the CeCILL license and can be freely downloaded from http://alcovna.genouest.org/mapsembler/.

## Background

Genomics witnessed an unprecedentedly deep change a few years ago with the arrival of the Next Generation Sequencers (NGS) also known as High Throughput Sequencing (HTS). These technologies enable sequencing of biological material (DNA and RNA) at much higher throughput and at cost that is now affordable to most academic labs. These new technologies generate gigabyte- or terabyte-scale datasets. The size of datasets is one of the two main bottlenecks for NGS. The other bottleneck is the analysis of generated data. Current technologies cannot output the entire sequence of a DNA molecule, instead they return small sequence fragments (*reads*) of length around a few hundred base pairs. Without a reference genome, reconstructing the entire sequence from these fragments (*de novo assembly* process) is challenging, especially in terms of computational resources. For instance, whole genome assembly of sequencing data from a mammalian genome requires hundreds of gigabytes of memory and several CPU weeks of computation [1-3].

With sequencing costs falling, sequencing efforts are no longer limited to the main species of interest (human and other primates, mouse, rat, *E. coli*, yeast, drosophila, …). Thus, biologists are increasingly working on data for which they do not have any close reference genome. In such situations, *de novo* assembly of reads is often carried out as a preliminary step. However, complete assembly is not always feasible, either because sequencing data is not adequate (insufficient coverage, genome too complex or many genomes present) or computational resources are too costly. Moreover, it should also be noted that assembly algorithms perform heuristics that lead to suboptimal reconstruction of the original sequence, possibly generating incomplete or erroneous fragments [2,3]. Especially, highly-similar occurrences of a repeated sequence can be collapsed into a single fragment.

We seek to establish that many biological questions can be answered by analyzing unassembled reads. In particular, the user may possess *a priori* information on which he wants to focus. In this spirit, we present the Mapsembler software. Mapsembler checks if a known piece of information - a sequence fragment called a *starter* - is present with a bounded number of substitutions in a set of reads. The starter can be shorter, longer or equal to the read length. If the starter is indeed present, Mapsembler constructs an assembly around the starter, either as a plain sequence, or as a graph showing divergences and convergences in the neighborhood structure. The read coverage per position is provided. The aim of Mapsembler is not to produce contigs as long as possible, hence it should not be used as a *de novo* assembler nor be directly compared with such software. Its aim, after the detection of the presence of approximate occurrences of a starter, is to output their neighborhoods on some hundreds or thousands of nucleotides, providing pieces of information about the starter context(s). As presented in the results section, these *micro* targeted assemblies provide relevant biological information such as the occurrences of elements known to be repeated, SNPs, gene fusions, alternative splicing events…

Mapsembler includes a simple yet effective error correction step removing most substitution errors present in the reads. As insertions and deletion errors are not corrected, Mapsembler performs better on reads provided by technologies generating a small amount of such errors as Illumina technology for instance.

Another key aspect of Mapsembler is that its memory usage is independent from the size of the read sets. This enables Mapsembler to analyze huge sets of reads on a simple desktop computer.

Mapsembler inputs are a sequence fragment or a set of fragments called the *starter(s)*, and a set of reads. Applications of Mapsembler cover a broad range of biological questions, including but not limited to: 

• For a known biological event, e.g. a SNP (*), a splicing event (*) or a gene fusion (*), Mapsembler can be used to check its presence in a set of reads, and to provide abundances in each case. This is done by using as starter a fragment localizing the event.

• Do these genes have close homologs in this set of reads (*)? Similarly, do these enzymes exist in this metagenomic set, or do these exons expressed in this [meta]transcriptomic set? Using genes or the enzymes or exons as starters, Mapsembler detects their presence and their approximate copies, and also reconstructs the genomic context for each copy. The exact coverage per position is provided both for the copies and for their contexts.

• In case of complex genomes, one may be interested in finding approximate repeated occurrences of known sequence fragments (*). Using such sequence fragments as starters, their occurrences within a fixed Hamming distance are found and their flanking regions are recovered as a graph.Note that this approach is limited to a small number of slightly differing occurrences. Indeed, graph-based Mapsembler results are mainly designed to be visually inspected.

•Mapsembler can be used to detect all reads corresponding to known contaminant organelles, or symbionts. This enables for instance to remove such reads from a dataset before further analysis.

The symbol (*) indicates that an example of this use case is given in the Results section. Furthermore, it is important to note that Mapsembler operates without a reference genome.

## Methods

The Mapsembler algorithm can be divided into two main phases: 

1. **Mapping**. Mapsembler detects which starters correspond to consensuses of reads, subject to coverage constraints and up to a bounded number of substitutions. Such starters are said to be read coherent (see Section “Sub-starter generation and read coherence”).

2. **De novo assembly**. Each read coherent starter is extended in both directions. In accordance to user choice: 

(a) the extension process is stopped as soon as several divergent extensions are detected. In this case, the output is a FASTA file containing the consensus assembly around each starter;

(b) the extension process continues even in the case of several divergent possibilities. Extensions are represented as a directed graph. Each node stores a sequence fragment and its read coverage per position. This graph, is output in *xgmml* or *graphml* format. Several tools, including Gephi [4], Cytoscape [5], and Cobweb [6] can be used to display such graph formats.

Mapsembler presents the advantage of not indexing reads but only starters (see next section for algorithmic explanations). In practice, independently of the size of the read file (even terabyte-sized), it is possible to run Mapsembler on any desktop or laptop computer, not requiring large memory facilities.

**Figure 1:**
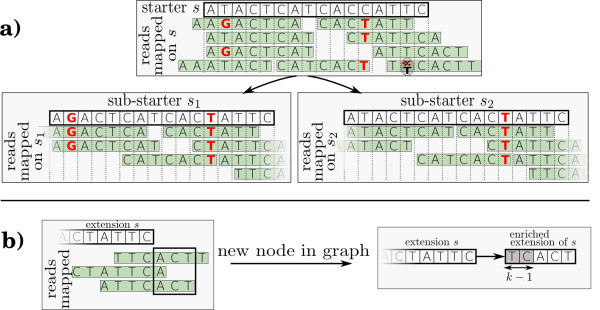
**Algorithm overview.** Overview of the algorithm steps with reads of length 7, a minimal coverage of 2 and k-mers of length *k*=3. **a)** Representation of the sub-starter generation step. A set of reads is mapped to the starter *s*. First, reads are error-corrected according to a voting procedure (see lower right read for instance). Then, each sub-starter (*s*_1 _ and *s*_2_) is computed from each perfect multiple read alignment. The Hamming distance between each sub-starter and *s* is required to be below a certain threshold. **b)** Representation of an extension. Three reads have prefix of length at least *k* mapping perfectly to the suffix of an extension *s*. All fragments of these reads longer than extension *s* are used for generating extension of *s*. As minimal coverage is 2, the last character of the first extending reads (*T*) is not stored for generating extension of *s*. The generated extension of *s* (*ACT*) is stored in a new node linked to extension *s*. Note that suffix of length *k*−1 of extension *s* (*TC*) is stored as prefix of extension of *s* (then called enriched extension). This avoids to omit overlapping *k*-mers between extensions such as *TCA* or *CAC* while mapping reads on extension of *s*.

The mapping phase performs several tasks. A maximum number *d*≥0 of substitutions (Hamming distance) is authorized between a starter and each read. Consequently, for a single starter *s*, several distinct read subsets that align to *s* yield distinct consensus sequences. These sequences are called sub-starters (see Section “Sub-starter generation and read coherence” for a formal definition), see Figure 1a for an example. We discard sub-starters for which the distance to *s* exceeds *d*. A local assembly is initiated from the extremities of each sub-starter.

### Definitions

We first introduce some notations and definitions used throughout the paper. A sequence ∈*Σ*^∗^ is a concatenation of zero or more characters from an alphabet *Σ*. A sequence *s* of length *n* on *Σ * is represented by *s*[0]*s*[1]…*s*[*n*−1], where *s*[*i*]∈ *Σ * ∀ 0≤*i*<*n*(if *i*<0 or *i*≥*n* then *s*[*i*]=*ε*, the empty string). We denote by *s*[*i*,*j*] (*j*≥*i*) the sequence *s*[*i*]*s*[*i* + 1]…*s*[*j*] of *s*. The sequence *s*[*i*,*j*] occurs position *i* in *s*. Its length, denoted by [*s*[*i*,*j*]], is equal to *j*−*i* + 1. The Hamming distance *d*_*H*_(*ω*_1_,*ω*_2_) between two sequences *ω*_1 _ and *ω*_2 _ of equal length is the number of positions at which the corresponding characters are different:

(1) $$d_{H}(\omega_{1},\omega_{2})=\overset{i<|\omega_{1}|}{\underset{i=0}{\sum }}\left\{\begin{array}{c} 1\;\text{if}\;\omega_{1}[\;i]\neq \omega_{2}[\;i]\\ 0\;\text{else}\; \end{array}\right).$$

#### Definition 1 (***Hamming distance for overlapping sequences***)

Given two sequences *ω*_1_ and *ω*_2_∈*Σ*^∗^, and i∈Z, we define *d*_*H*_(*ω*_1_,*i*,*ω*_2_) as the hamming distance of the overlapping part between *ω*_1_ and *ω*_2_, considering the first character of *ω*_2_ aligned to position *i* on *ω*_1_. Formally, $d_{H}(\omega_{1},i,\omega_{2})=\overset{j<|\omega_{2}|}{\underset{j=0}{\sum }}d\left(\omega_{1}\left[i+j\right],\omega_{2}[j]\right)$, where $d(\alpha ,\beta )=\left\{\begin{array}{c} 0\;\text{if}\;\alpha \;=\;\beta \;\text{or}\;\alpha =\varepsilon ,\\ 1\;\text{otherwise} \end{array}\right)$. The character *ω*_1_[*i* + *j*] is equal to *ε*if a prefix of *ω*_2 _ is not aligned with *ω*_1_(*i* + *j*<0) and/or if a suffix of *ω*_2 _ is not aligned with *ω*_1_(*i* + *j*≥|*ω*_1_|).

#### Definition 2 (***Mapped read***)

Given a sequence *s*∈Σ^∗^, a read *r*∈Σ^∗ ^ is said to be mapped to *s* at position *i iff d*_*H*_(*s*,*i*,*r*)≤*d*, where *d* is a fixed threshold.

The notation $s{∥}_{i}^{d}r$ denotes that *r* maps on *s* at position *i*, with threshold *d*.

#### Example 1 (***Mapped read***)

Given *s*=*ATTCGGA*, *r*=*GAATGCG* and threshold *d*=1, $s{∥}_{-2}^{1}r$ is true as *d*_*H*_(*s*,−2,*r*)=1:

(2) $$\begin{array}{lllllllll} -2 & -1 & 0 & 1 & 2 & 3 & 4 & 5 & 6\\ s= & A & T & G & C & G & G & A\\ | & | & \cdot & | & |\\ r= & G & A & A & T & T & C & G \end{array}$$

### Algorithm

An overview of the whole process is presented in Algorithm 1. In a few words, the algorithm is divided into two main phases: the **mapping phase** (Steps 1 to 4 of Algorithm 1). This first phase is similar to seed-based mapping algorithms such as Gassst[7]. However, sub-starter generation (Step 4) is a novel algorithm presented in Section “Sub-starter generation and read coherence”. The second phase is the **targeted*de novo*assembly phase** (Steps 6 to 14). This phase extends sub-starter sequences similarly to greedy *de novo* assembly algorithms, such as SSAKE [8]. Extensions are stored in a graph using a novel procedure (Steps 12 and 15) presented in Section “Graph management”.

### Algorithm 1: Mapsembler overview

**Requires:** Set of reads *R*, set of starters *S*, integer value *k*; **Ensure:** For each starter in *S*, the sub-starters and extensions 

1: Index the *k*-mers of *S*

2: Map reads *R* to each starter from *S*, using the *k*-mer index

3: **for all***s * ∈ *S ***do**

4: Using reads mapped to *s*, generate sub-startersof *s*.

5: Add new sub-starters to *Ext*_0_.

6: *i*=0

7: **while***Ext*_*i*_≠*∅***do**

8: Free previous index, index *Ext*_*i *_ with *k*-mers

9: Map reads *R* to sequences of *Ext*_*i*_, using the*k*-mer index

10: **for all***s * ∈ * Ext*_*i*_**do**

11: Using reads mapped to *s*, generate extensions of *s*.

12: Create nodes containing the extensions &manage graph

13: Store all novel extensions in *Ext*_*i* + 1_

14: *i * = * i * + 1

15: Simplify the created graphs

16: For each starter in *S*, output its sub-starters and their extensions

### Explanation of Algorithm 1 steps

• Step 1: An index of all *k*-mers that appear in the initial starter set *S* is created. For each sequence *s*_*id *_ belonging to the indexed set, and for each *k*-mer in *s*_*id*_, a list of couples (*s*_*id*_,*p*_*id*_) is stored, with *p*_*id *_ being a position where the *k*-mer occurs in *s*_*id*_. Note that, as a *k*-mer may occur more than once in a sequence *s*_*id*_, several distinct couples may be stored for a given *k*-mer and a given *s*_*id*_. All couples (*s*_*id*_,*p*_*id*_) of a given *k*-mer can be accessed in constant time using a hash table with the *k*-mer as key.

• Step 2: input reads (and their reverse complement) are processed on the fly, only mapped reads are stored in memory. The mapping process is as follows. All *k*-mers of each read are used as seeds to attempt to map the read to the indexed sequences. After the entire set of reads is processed, an error correction step (described in the next paragraph) removes sequencing errors from mapped reads. Each error-corrected mapped read *r* (∃*i*such that $s{∥}_{i}^{d}r$) is stored in the set $M_{s}$.

• Steps 8 and 9: Indexing of extensions *Ext*_*i *_ and read mapping are performed similarly to Steps 1 and 2. During these steps, reads have to perfectly agree with the extensions, hence read mapping is done with distance threshold *d*=0.

• Step 11: For each sub-starter, extensions are always stored in a rooted directed string graph, each node containing a sequence fragment. A node storing a sequence *s* is denoted by *N*_*s*_. The node storing the sub-starter itself is the root of the graph. For each sequence *s * ∈ * Ext*_*i*_, using all error-corrected mapped reads $M_{s}$, detect those whose suffix stops after *s* ends (see Figure 1b for an example). Those reads are used to compute the extension(s) of *s*, yielding three cases: 

1. An empty extension is found.

2. Exactly one extension *e* is larger than *s*. Create a node *N*_*e*_, and link the node *N*_*s *_ to the node *N*_*e*_. Store the fragment *e* in *Ext*_*i* + 1_.

3. Several extensions {*e*_1_,*e*_2_,…,*e*_*n*_} are found, then: 

For simple sequence output, the longest common prefix *p* of all *e*_*i *_ is stored in a new node *N*_*p*_. Link *N*_*s *_ to *N*_*p *_ for output purpose. As *p* is not stored in *Ext*_*i* + 1_, its extension stops.

For graph output, link *N*_*s *_ to *n* new extending nodes each storing an extending fragment. All fragments in {*e*_1_,*e*_2_,…,*e*_*n*_} are stored in *Ext*_*i* + 1_.

• Step 12: Generate enriched extensions by adding suffix of *s* of length *k*−1 as prefix of each extension of *s* (see Figure 1b). By adding such a prefix, we ensure that each node stores a sequence long enough (at least *k*) to be indexed and then exploited for next extensions and that each *k*-mer, including those overlapping nodes are considered as seeds.

Step 13: Novel extensions are those corresponding to nodes which are not already present in the graph (see Section “Graph management”).

Step 16: In case of simple sequence format, the extensions graph of each sub-starter do not contain branching nodes. A simple traversal provides the consensus sequence of the contig containing the sub-starter.

#### Error correction

Actual sequencing reads are error-prone, therefore error correction mechanisms are implemented inside the mapping phase. At Steps 2 and 9, error-prone reads are mapped to starters. An error correction phase is performed immediately after both of these steps, by taking advantage of the multiple read alignments. This procedure is based on nucleotide votes, similarly to greedy assemblers [8], under the assumption that erroneous nucleotides are less represented than correct nucleotides. Specifically, at each position relative to the starter, the count of each nucleotide is recorded. Given a threshold *t*, a read position is considered to be correct if the corresponding nucleotide at this position is seen at least *t* times. Otherwise, if only one other nucleotide appears over *t* times at this position, the read position is corrected by assigning this other nucleotide (Figure 1a, circled position). In the remaining case, where many possible nucleotides can possibly correct a read position, no correction occurs, and the read is truncated before this position.

We now provide deeper algorithmic explanations for sub-starter generation (Step 4) and the graph management (Steps 12 and 15). The remaining steps (read mapping and greedy sequence extensions) are classically well known [7,8].

### Sub-starter generation and read coherence

The sub-starter generation and read coherence step take place immediately after the mapping phase (Step 4). Given a starter *s* and mapped reads *R*, this step generates a finite set (*s*_*i*_) of sequences (called *sub-starters*) which: 

originate from the reads, i.e. each *s*_*i*_ is a consensus sequence of a subset of reads from *R*,

are coherent with the starter *s*, i.e. the Hamming distance between *s* and *s*_*i *_ is at most *d*.

are significantly represented, i.e. each position of *s*_*i *_ is covered by at least *c* reads.

A starter is *read coherent* if it yields at least one sub-starter. We are interested in retrieving the largest set of sub-starters for each starter *s*. This can be formulated as the following computational problem. To simplify the presentation, reads are assumed to contain no errors. In practice, the read correction step (previous paragraph) effectively corrects or discards erroneous reads.

#### Problem 1 (***Multiple consensuses from read alignments***)

Given a starter *s*, two parameters *c*, * d * ≥ 0 and a set of error-free mapped reads $R=\{r_{i}\;\text{such that s}{∥}_{p_{i}}^{d}r_{i}\}$ (each read *r*_*i *_ is aligned to *s* at a position *p*_*i *_ with at most *d* substitutions), find all maximal (with respect to the inclusion order) subsets *S*_*i *_ of *R* satisfying: 

1. each subset *S*_*i *_ admits a perfect consensus *s*_*i*_, i.e. each read *r*_*i *_ aligns to *s*_*i *_ at position *p*_*i *_ (relative to *s*) with no mismatch: $s_{i}{∥}_{p_{i}}^{0}r_{i}$,

2. the consensus *s*_*i *_ aligns *s* with at most *d* mismatches: $s{∥}_{0}^{d}s_{i}$,

3. each position of *s* is covered by at least *c* reads in *S*_*i*_.

A trivial (exponential) solution is (i) to generate the power set (all possible subsets) of *R*, (ii) remove sets which do no satisfy one of the propositions above, and (iii) keep only maximal sets (ordered by inclusion). The exponential complexity of this solution clearly comes from step (i). In Algorithm 2, we give a polynomial time (in the number of mapped reads) procedure which subsumes (i), as it generates a solution which includes all the correct subsets.

The completeness proof that Algorithm 2 finds all maximal subsets corresponding to correct sub-starters is as follows. The proof is by contradiction: let *s* be a correct sub-starter not found by the algorithm. Let *r*_1_,…,*r*_*n*_ be the maximal subset of reads which yields *s*, sorted by increasing mapping positions to *f*. We show by induction that the algorithm returns a subset which includes *r*_1_,…,*r*_*k*_, for *k*∈[1*.n*]. For *k*=1, notice that a subset is assigned to each read. Assuming *r*_1_,…,*r*_*k *_ is part of a returned subset *S*_0_, we show that *r*_1_,…,*r*_*k* + 1_ is also returned. Since *r*_*k* + 1_ is part of a subset which yields *s*, it overlaps perfectly with *r*_*k*_. However, *r*_*k* + 1_ does not necessarily belong to *S*_0_. Let $r_{k+1}^{\prime }$ be the read which follows *r*_*k*_ in *S*_0_. In the ordering of the reads by increasing position, if the read *r*_*k* + 1_is seen before $r_{k+1}^{\prime }$, then the algorithm selects $r_{k+1}^{\prime }=r_{k+1}$. Else, as *r*_*k* + 1_ perfectly overlaps with *r*_*k*_, a new subset is created from *S*_0_, which contains exactly *r*_1_,…,*r*_*k* + 1_. Eventually, from the induction, a subset which contains *r*_1_,…,*r*_*n*_ is constructed. Since *r*_1_,…,*r*_*n*_ is itself maximal, the subset found by the algorithm is exactly *r*_1_,…,*r*_*n*_.

Note that Algorithm 2 may return subsets which do not satisfy all the three conditions (e.g. coverage of *s* after the last aligned read position *p* is not checked), hence steps (ii) and (iii) are still required. The running time of the algorithm is now analyzed. Observe that during the algorithm execution, each intermediate subset in *S* is included in a distinct final maximal subset. There are at most |*Σ*|^*d*^ maximal subsets, one for each combination of substitutions with *s*. Hence, there are *O*(|*Σ*|^*d*^) intermediate subsets at any time. Assuming that the read length is bounded by a constant, the overlap detection steps 4 and 7 can be performed in *O*(|*R*|) time. Hence, the time complexity of Algorithm 2 is *O*(|*Σ*|^*d*^|*R*|^2^), where in practice *d* is a small constant, and |*Σ*|=4 on genomic sequences.

### Algorithm 2: Generating candidate subsets *S*_*i *_ for solving the multiple consensuses from read alignments problem

**Requires:** Set of reads *R*, starter *s*, minimum consensus *c*≥0, distance threshold *d*≥0; **Ensure:** Set *S* of candidate subsets. 

1: *S*=*∅*.

2: **for** each read (*r*,*p*) in *R* ordered by alignment position **do**

3: **for** each subset *S*_*i *_ in *S***do**

4: **if** r overlaps without substitutions with the lastread of *S*_*i *_**then**

5: Add *r* to *S*_*i*_.

6: **else**

7: **if***r* overlaps without substitutions with oneof the reads of *S*_*i *_**then**

8: Let (*r*^*′*^, *p*^*′*^) be the last read of *S*_*i *_ overlapping with *r*.

9: Let *T* be the subset of *S*_*i*_ of all reads up to(*r*^*′*^,*p*^*′*^).

10: Create a new subset *S*^*′*^=*T*∪{*r*}.

11: Insert *S*^*′*^ into *S*.

12: **if***r* was not appended to any subset **then**

13: Create a new subset with *r* and insert it into *S*.

14: Remove any subset from *S* if its consensus hasmore than *d* differences with *s*, or a position before *p* is covered by less than *c* reads.

15: **return ***S*.

### Graph management

#### Adding a node

Several biological events such as a SNP, an indel, or exon skipping, create two or more distinct paths in the extension graph. These paths eventually converge and continue with an identical sequence. Consequently, path convergence is checked during the iterative assembly phase (Algorithm 1, Step 12). When a sequence *s* is extended with extension *e*, the algorithm checks if *e* is not already present in the graph in a node $N_{s^{\prime }}$. To do this, the last *k*-mer of the sequence of each node is indexed in a hash table. Checking if *e* is already present in the graph is done using *k*-mers of *e* and this last index as seeds for mapping. If the overlap of *e* on the sequence of a node $N_{s^{\prime }}$ is perfect (*i.e.*$\exists i,\;{\text{such that s}}^{\prime }{∥}_{i}^{0}e$) then *N*_*s*_ is linked to $N_{s^{\prime }}$. If *i*<0, an intermediate node containing the prefix of *e* not mapped on *s*^*′*^ is added between *N*_*s*_ and $N_{s^{\prime }}$. If *i*>0, the suffix of length *i* of *s* is pruned from node *N*_*s*_ as it is already present in node $N_{s^{\prime }}$.

#### Graph simplification

**Figure 2:**
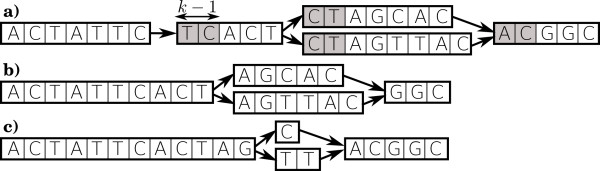
**Graph simplification.** Graph simplification (Algorithm 1, Step 15). **a)** the graph before simplification. **b)** After removing the first *k*−1 characters of each internal node and after merging non branching nodes. **c)** After common prefix and suffix factorizations.

Once extensions are finished, each graph is simplified as follows: 

As presented in Figure 2a-b, enriched extensions are transformed into extensions, by removing the first *k*−1 characters of each internal node except the root. This removes redundant information in nodes.

Two nodes *N*_*s*_ and $N_{s}^{\prime }$ are merged into node $\text{Ns.}.s^{\prime }$ if and only if *N*_*s*_ has only $N_{s^{\prime }}$ as successor while $N_{s^{\prime }}$ has only *N*_*s*_ as predecessor. This is a classical concatenation of simple paths. See Figure 2a-b for an example.

For all nodes successors of a node *N*_*s*_ having only *N*_*s*_ as predecessor, their longest common prefix *pre* is pruned and factorized as suffix of the sequence stored in *N*_*s*_, thus generating node *N*_*s.pre*_. Similarly, for all nodes predecessors of a node *N*_*s*_ having only *N*_*s*_ as successor, their longest common suffix *suf* is pruned and factorized as prefix of the sequence stored in *N*_*s*_, thus generating node *N*_*suf.s*_. This simplification relocates branching in the graph, to the exact position where sequences diverge and converge. See Figure 2c for an example.

## Availability and requirements

Mapsembler is released under CeCILL License. It can be downloaded from http://alcovna.genouest.org/mapsembler/website. The download comes with documentation about installation and usage. A Mapsembler newsletter is also available from this address.

## Results

All presented results were obtained on a 2.66 Ghz dual-core laptop with 3 MB cache and 4 GB RAM memory.

For each experiments presented in this manuscript, details about datasets, Mapsembler commands and results are packed in Additional file 1. When read datasets are public, a link to a download address is provided in the archive. When they are not, only reads used by Mapsembler during mapping and assembly phases are provided.

In Figures representing graphs, the node size indicates average read coverage in the sequence and the node border size indicates the length of the sequence.

Note that Mapsembler is not designed to be a whole genome assembler, thus classical assembly statistics (N50, genome coverage, …) do not apply. Apart from nucleotide accuracy and rate of misjoins, quality measures for *de novo* targeted genome assembly are, to the best of our knowledge, not defined.

### Mapsembler and the state of the art

Targeted assembly should not be confused with Sanger-generation, localized BAC-by-BAC assembly methods (e.g. Atlas [9]). BAC-by-BAC sequencing is typically not performed anymore in second-generation sequencing. Mapsembler computes targeted assemblies within a whole-genome set of short reads, i.e. without any localized sequencing process. To date, we are aware of only one related targeted assembly method in the literature, Tasr[10].

Tasr is based on the Ssake assembler [8]. It maps a set of reads on targets (starters) using seeds of length 15. Mapping between a read and a target is tested if at least one sequence of length 15 exactly matches both. Tasr outputs the result of this mapping, including extensions obtained from reads mapped to extremities of starters. Similarly to Mapsembler, Tasr indexes only targets, hence memory requirements do not depend on the size of the read file. Mapsembler significantly differs from Tasr as it offers the following novel features: 

sub-starters retrieval;

multiple iterations to extend starters as far as possible. This is equivalent to re-running Tasr multiple times, using its results as starters;

graph output of the left and right neighborhood of starters.

We compared Tasr and Mapsembler time and memory performances using a set of 6.5 millions of short reads of length 36 (unpublished Illumina aphid RNA-seq). We ran Mapsembler without iterative extensions and set seeds length to 15, to match Tasr behavior.

Using a unique randomly selected read as starter, Mapsembler finished in 40 seconds, using 2.15 MB of memory, while Tasr finished in 165 seconds using 4.21 MB of memory. On a larger set of 500 starters randomly selected from reads, Mapsembler finished in 59 seconds using 23.8 MB of memory. Tasr was stopped after 10 hours, while using 287 MB of memory. Note that in both cases, Mapsembler produces strictly more results than Tasr as it detects and extends all the sub-starters of each starter.

The iterative mapping and assembly strategies are also used in the IMAGE approach [11], although in a different context. IMAGE maps paired-end reads to a pre-assembled set of contigs in order to extend contig lengths and close gaps. Mapsembler could theoretically be used to extend contigs with unpaired reads, but does not perform automated gap closing. In practice Mapsembler is an orthogonal approach to IMAGE, as it aims to replace whole-genome assembly for a subset of biological questions.

### Assembly accuracy

The accuracy of Mapsembler targeted assemblies is assessed. We performed targeted assembly of 50 starters of length 37 nt sampled uniformly from the *E. coli* genome. These starters were assembled using 20.8 M raw Illumina reads (SRA: SRX000429). Mapsembler was run with default parameters and *d*=0, to discard sub-starters which do not correspond exactly to starters. Using 40 iterations, Mapsembler returned 50 extended sub-starters of average length 812 nt. We computed global alignments between Mapsembler extensions and the reference genome. For each alignment, the reported accuracy corresponds to the ratio of the number of substitutions and mismatches over the number aligned bases. Each targeted assembly aligns with more than 99% accuracy, and no misjoin was produced. Specifically, 97.8% of the extensions were perfectly aligned. This level of accuracy is consistent with that of whole-genome *de novo* assemblers. For instance, 96.5% of the contigs from a SOAPdenovo [12] whole-genome assembly of the same dataset align perfectly to the reference.

### Dealing with large data sets

In this section, we focus exclusively on Mapsembler time and memory requirements. From the NCBI Sequence Read Archive, we downloaded a human NA12878 Illumina run containing 105 million reads of length 101 (SRR068330, total 10.6 Gbases). Five subsets, *S*_10*K*_, *S*_100*K*_, *S*_1*M*_, *S*_10*M*_, and *S*_100*M*_, were generated by random sampling of 10^5^, 10^6^, 10^7^, 10^8^ and 10^9^ reads. A targeted assembly of 10 randomly selected reads as starters was performed using Mapsembler with default options.

Results summarized in Table 1 show that memory requirement does not depend on the read file size. Note that a read file containing 9.9 Gbases (*S*_100*M*_ data set) was analyzed using <1.5 MB of memory. These results also show that computation time is reasonable even on such large data sets as time linearly increases with the number of starters. On average on the *S*_100*M*_ data set, checking read coherence of all starters took 1813 seconds while one extension of all sequence fragments took 903 seconds. Mapsembler computation time grows linearly with respect to the number of input starters and the number of computed extensions. Note that an option enables to limit the number of extensions, and note that if manually stopped, Mapsembler outputs results obtained so far.

**Table 1:** Mapsembler time and memory requirements on large data-sets

**Reads data set**	**Mapping time (s)**	**Assembly time (s)**	**Total time (s)**	**Memory (MB)**
*S*_10*K*_	<1	<1	1	<1.5
*S*_100*K*_	1	2	5	<1.5
*S*_1*M*_	14	6	40	<1.5
*S*_10*M*_	170	95	442	<1.5
*S*_100*M*_	1813	903	3983	<1.5

Time and memory requirements for targeted assembly of 10 starters using increasingly large human genome read data sets. Mapping time corresponds to the mapping phase (Algorithm 1, Steps 1 to 5). Assembly time corresponds to the assembly phase (Steps 18 to 14) per iteration.

### Recovering environments of repeat occurrences

**Figure 3:**
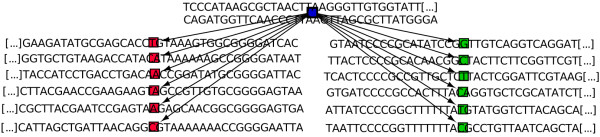
**Repeated starter.** Graph obtained using a repeat occurrence as starter. To be readable the prefixes of left extensions and the suffixes of right extensions, as well as the core or the starter are truncated.

We analyzed a dataset of 20.8 M raw Illumina reads (SRA: SRX000429) from *E. coli K12* using as starter a sub-sequence of the reference genome (chr:15,387-16,731) containing Inserted Sequences IS186 and IS421 transposase. This fragment has six exact occurrences on the reference genome. Using 3 iterations, Mapsembler needed 202 seconds and 1.5 MB of memory to produce the graph presented in Figure 3. The graph yields neighbor sequences of all occurrences of this repeat. The six occurrences were exactly recovered by Mapsembler. Where classical whole genome assemblers interrupt an assembly, Mapsembler retrieves the environments of the occurrences of a repeat. In this case, the exact number of occurrences can be directly inferred from Mapsembler graph structure.

### Detecting AluY sub-families in a personal genome

*Alu* elements are a family of highly-repeated, ≈300 bp sequences found in primate genomes. Sub-families of the AluY family are characterized by known evolutionary mutations. We demonstrate how Mapsembler can be used to detect the AluY sub-families present in a set of reads.

We downloaded a dataset of high-coverage, NA12878 chromosome 19 reads from the 1000 Genomes project. We selected bases 60-120 of the RepBase [13] consensus sequence of AluY as a starter, as prior knowledge indicates that no indel occurs inside this region. Mapsembler then processed on the whole dataset (65 M reads) to recover sub-starters, without extending them. Mapsembler error and coverage thresholds were increased according to the coverage of the dataset, and 5 substitutions were allowed between the starter and each sub-starter.

A total of 58,656 reads mapped to the 60 bp starter and 8 sub-starters were constructed by Mapsembler. We examine the specificity of Mapsembler by verifying that sub-starters correspond to known consensus sequences. We annotated each sub-starter using sub-families consensus sequences [14] and the NA12878 reference sequence [15].starter TCACGAGGTCAGGAGATCGAGACCATCCTGGCTAACACGGTGAAACCCCGTCTCTACTAA AluY substarter_0 ---CG---------------------------C---------------CG---------- AluSgsubstarter_1 ---CA---------------------------T---------------CA---------- substarter_2 ---CG---------------------------T---------------CA---------- chr19_maternal 517189substarter_3 ---TG---------------------------T---------------CA---------- substarter_4 ---CG---------------------------T---------------TG---------- chr19_maternal 887598substarter_5 ---CG---------------------------T---------------CG---------- AluY (starter)substarter_6 ---CA---------------------------T---------------CG---------- chr19_maternal 461151substarter_7 ---TG---------------------------T---------------CG---------- AluYb8

Several sub-starters (1, 2, 3, 4 and 6) did not exactly correspond to a known Alu consensus sequence. We manually verified that all these sub-starters are valid as follows. Sub-starters 2, 4, 5 and 6 (resp. 2, 4 and 5) align perfectly to the NA12878 maternal (resp. paternal) reference. Mutations of sub-starters 2 and 4 (bases 50 and 49 respectively) are also found in Alu Ya5 [16]. As further evidence, sub-sequences specific to each substarters (bases 3 to 50) are abundantly present as exact substrings in the reads. For instance, bases 3 to 50 of the remaining unidentified sub-starters (1 and 3) are present in respectively 73 and 76 reads. Consequently, the possibility that sub-starters 1 and 3 are artifacts was ruled out.

### Gene detection in a different strain

The folA gene (dihydrofolate reductase) is present in several strains of *E. coli*, including *K12* (chr:49,823-50,302) and O157:H7 (chr:54,238-54,717). The sequence of this gene is not exactly similar between the K-12 and O157:H7 strains (10 single-nucleotide mutations across 479 bp). We attempted to recover the O157:H7 gene sequence of the folA gene, using only sequencing reads and prior knowledge of the K-12 sequence. To this end, we analyzed a dataset of 15.7 M raw reads of length 70 bp (SRA:ERR018562) from *E. coli O157:H7*. The K-12 allele of the folA gene (length 479 bp, NCBI ID:944790) was used as the starter. The sub-starter generation module of Mapsembler confirmed the presence of the gene, and furthermore recovered the exact O157:H7 gene sequence of folA from the reads (100% identity with O157:H7 reference). Mapsembler performed this experiment in 572 seconds and using 1.5 MB of memory.

### Detection of known biological events in *Drosophila*

In this section, one Illumina HiSeq2000 RNA-Seq run of 22.5 million reads of length 70 nt from Drosophila Melanogaster is analyzed (data not published). As presented in upcoming sections, Mapsembler enables to check for the presence or absence of a putative biological event for which one has an a priori knowledge, and to provide additional information in case of presence. Recall that the tool is not dedicated for calling blindly all such events in a high throughput sequencing dataset.

#### Exon skipping

**Figure 4:**
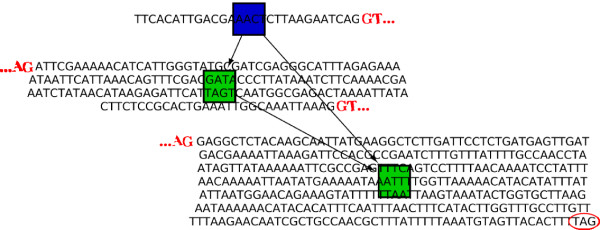
**Drosophila exon.** Visualization of Mapsembler results on a drosophila read data set. Red characters correspond to splice sites found by mapping using Blat [17], while the circled characters is a codon stop.

**Figure 5:**
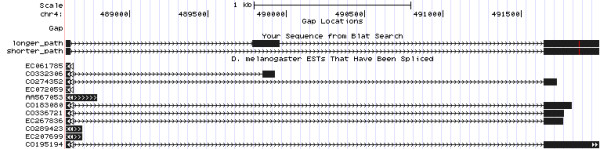
**Drosophila exon - blat result.** Visualization of Blat results on sequences obtained from graph presented Figure 4. Shorter path corresponds to the concatenation of the sequences from starter node (blue node) and from the lowest node, while longer path corresponds to the concatenation of the sequences from starter node, left most node and lowest node. The central node includes, but is not limited to a known EST CO332306.

We chose a starter located close to a known exon fragment (Chr4:488,592-488,620 BDGP R5/dm3). Using less than one megabyte of memory and in 33 minutes, Mapsembler confirmed the presence of this exon fragment. The corresponding part of the obtained graph is presented in Figure 4, while a visualization of the Blat [17] result is presented Figure 5.

#### Visualizing SNPs

**Figure 6:**
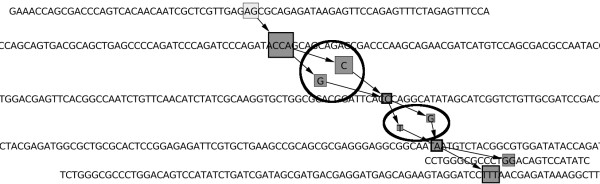
**Drosophila SNPs.** Visualization of Mapsembler results on a drosophila read data sets, looking for known SNPs. On this graph, 2 SNPs (circled nodes) in the right extensions are shown. Full sequences are truncated.

On the same read data set, we used a fragment (chrX:17,783,737-17,783,812 BDGP R5/dm3) for which neighboring genes are known. We applied Mapsembler using this fragment as starter and obtained results in less than 1 megabyte of memory and less than 110 minutes of execution (40 iterations). The results presented in Figure 6 enable to visualize the SNPs. Note that these results do not bring phasing SNP information.

### Detection of fusion genes in breast cancer

Recent work from Edgren *et. al.*[18] uses paired reads from RNA-seq experiments (SRA: SRP003186) to detect fusion genes using a reference genome (Ensembl version 55). Mapsembler enabled to retrieve these fusion genes and enabled to detect new candidate fusion genes implicated in human breast cancer. Here, we present results for cell line BT-474, for which we downloaded the short reads used in [18] (merging runs SRR064438 and SRR064439). This data set contains ≈ 43 million reads of average length 51bp. Using extremities of fusions as starters, in 1 hour and 36 minutes, using ≈ 70 MB of memory, Mapsembler retrieved in 25 iterations fusions genes detected in [18].

**Figure 7:**
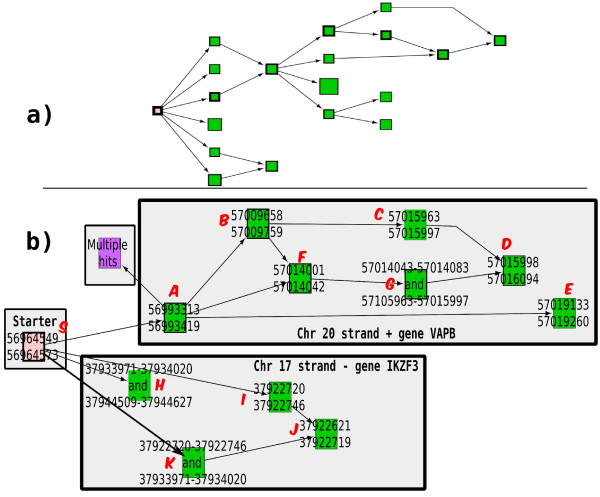
**Gene fusion in human breast cancer.** Extension graph of an extremity of an exon from the VAPB human gene located on chromosome 20. **a)**: the raw graph produced by Mapsembler. **b)**: the same graph manually curated by mapping the sequence of each node on the human genome. Nodes where moved in order to reflect their relative mapping position on the chromosomes. Nodes from the raw graph having sequences mapping at the same position where merged. For each node, the start and stop positions of the mapping are indicated. The presence of two start and stop positions reflects the presence of a central intron. Except for the purple node having multiple hits among the genome, 100% of the sequence of each node was mapped, either to an exon from gene VAPB on chromosome 20 or from gene IKZF3 on chromosome 17. The bold edge corresponds to the gene fusion found in [18], while the two other edges starting from the starter and targeting a chromosome 17 exon are new gene fusions.

**Figure 8:**
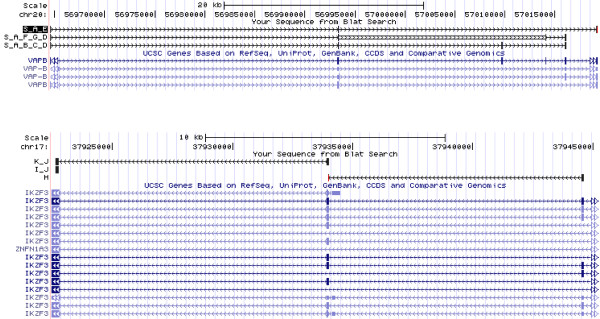
**Gene fusion in human breast cancer - Blat results.** Blat [17] results obtained after mapping paths from the starter to a leaf of the graph presented Figure 7. Succession of nodes of each mapped path (black lines) are indicated by their identifiers (red letters in Figure 7). Path belonging to gene VAPB chromosome 20 are represented on the upper part of the figure (S_A_B_C_D, S_A_F_G_D and S_A_E) while those belonging to gene IKZF3 on chromosome 20 (H, I_J and K_J) are represented on the lower part. Note that the starter is not mapped on gene IKZF3 as it appears only on chromosome 17 on the genome. However, it is concatenated to rightmost exons of each of the three paths (H, I_J and K_J) in the transcripts.

It is of particular interest to notice that Mapsembler retrieved these fusion genes without making use of a reference genome nor information between read pairs. As shown Figures 7 and 8, for junction *VAPB-IKZF3*, Mapsembler enabled to retrieve the fusion gene described in [18] and additionally detected two other fusions between genes *VAPB* and *IKZF3*, on different exons than those previously described. Moreover, as this is usually the case while applying Mapsembler on RNA-seq data, the graph output enables to retrieve the exon structure in the extensions.

## Discussion

We presented Mapsembler, a new tool for targeting specific pieces of information from a possible huge set of reads, on a simple desktop computer. Presented results show that such software has great potential for querying information from next generation sequencer reads. It enables to confirm the presence of a region of interest and retrieve information about surrounding sequence context. This approach presents the advantage to avoid a costly and approximative [2] full *de novo* assembly. However, Mapsembler presents some limitations discussed in this section.

### Homology/similarity distance

Mapsembler allows *d* substitutions between each starter its sub-starters. Hence, this homology distance is limited to a few percent of the starter length. Thus Mapsembler can not be used for searching homologous genes having less than, say, 90% of similarity.

Furthermore, setting a large *d* is not recommended for two reasons. Firstly, in the worst case, there are *O*(|*Σ*|^*d*^) sub-starters having at most *d* substitutions with a starter *s*. To avoid dealing with an arbitrarily large number of sub-starters, Mapsembler implements a limit of 100 sub-starters per starter.

Secondly, Mapsembler output sub-starters which may contain uncorrelated mutations, i.e. false positives. Consider a starter which contains two SNPs A/B and C/D sufficiently far away, so that are not spanned by any read. Mapsembler would reconstruct 4 sub-starters, corresponding to AC, AD, BC, and BD, even if only two of them were actually present in the sequenced organism.

### Paired reads vs. single reads

The Mapsembler algorithm does not use the paired reads information. Such information is difficult to incorporate in the iterative micro assembly process. We chose to discard it to keep the algorithm simple and applicable to any kind of data. However, in the case of the graph output, paired reads would enable to provide more information, for instance in splicing events. The graph constructed from single reads contains all possible junctions. Paired reads can be used to eliminate paths with two or more branching junctions which do not correspond to true isoforms.

Instead of injecting the paired read information in the algorithm, we believe that it is simpler to run Mapsembler, then use a third party algorithm (such as Blastree[19]) to map paired reads to the graph and output pairs-coherent paths.

### Micro assembly vs. full contigs assembly

One key aspect of Mapsembler is to be usable on a simple desktop computer, not requiring large memory facilities. This is achieved by an iterative algorithm which avoids indexing the read set. However, as shown Table 1, on common datasets generated by a run of an existing NGS platform, each iteration can take hundreds of seconds. Depending on the length of the reads, each iteration extends sub-starters by a few dozens nucleotides.

#### Does contig length matter?

If feasible on the data, using Mapsembler for the creation of contigs of length around 100kb or more would take weeks on a classical desktop computer. Consequently the user should specify a maximal number of iterations, or manually stop the process after a while.

We argue that short contigs provide sufficient biological information for our purpose. As presented in the results section, we retrieved SNPs, different isoforms and gene fusions using short contigs. For instance, the graph presented Figure 7, created in 1h36 after 25 iterations, stores a path of length 422 nucleotides. This whole graph is sufficient to detect the presence of 11 exons spread over two genes.

#### Sensitivity to SNPs

Similarly to greedy assemblers, in the simple sequence output mode, Mapsembler aborts sequence extension as soon as more than as one extension are found. This mechanism typically yields short neighbors, in particular in sequences containing SNPs. Thus we implemented an option to allow merging of multiple extensions having the same sequence except for one substitution. In this case, the substitution position is replaced by the nucleotide having the largest coverage. This effectively resolves ambiguities due to SNPs and generate longer extensions.

#### Starter selection

The input starters are sequence fragments on which reads will be mapped. They can be of any length, however very long starters (over 10^4^nt) are discouraged, as the sub-starters generation step is quadratic in the number of aligned reads. Furthermore, Mapsembler verifies that starters are read-coherent, hence longer starters are more likely to contain regions where the coverage is too low. Also, as previously mentioned, long starters may lead to false positive sub-starters.

Mapsembler discards read alignments which contain an indel. Hence, it is advised to input small, well-conserved starters. However, indels in the extensions are retained in the graph structure. Starters are typically constructed from an external source of information, such as sequence information from a related species, a known conserved gene, or an existing collapsed assembly.

#### Full biological events calling versus Mapsembler

Mapsembler was clearly not designed for calling broadly biological events. It should not be used for this purpose. Its usage should then be limited to cases where user has a piece of priori knowledge she wants to validate and extend, without making use of heavy and heuristics approaches.

## Conclusions

Mapsembler is a simple yet powerful, non-specific tool for extracting targeted pieces of information from newly sequenced, non-assembled genomes or transcriptomes. Technically, Mapsembler retrieves the approximate occurrences of a region of interest and performs targeted assembly through repeated iterations of read mapping. It also provides the possibility of visualizing the genomic context of assembled sequences as a graph. Mapsembler is not a whole-genome assembly software, instead it focuses on specific targets and assemble their contexts over a few hundreds of nucleotides. Mapsembler can be executed on a classical desktop computer; without cleaning the data and without a reference genome. Usage possibilities are numerous and fit the actual trend of sequencing, as more and more species, including meta-genomes, are sequenced without reference genomes.

We presented the main Mapsembler features and algorithmic ingredients. We have shown a selected overview of Mapsembler applications, among which one enabled to detect novel fusion genes. Benchmarks were ran on very large amounts of biologically relevant data. With respect to other comparable method, Mapsembler runs consistently faster and consumes less memory. More importantly, Mapsembler has several novel features, such as sub-starters retrieval, iterative extensions and graph visualization.

There is much room for future work. Currently the error correction is based on substitutions only. For opening Mapsembler to broader technologies like Roche 454 System, insertions and deletions will be taken into account during read correction.

To finish, its simplicity and its power make Mapsembler a good candidate for ambitious future NGS applications. In particular, even if it was not initially designed in this spirit, Mapsembler is highly parallelizable and can be adapted to a *“zero memory”* whole genome *de novo* assembly tool.

## Competing interests

The authors declare that they have no competing interests.

## Author’s contributions

PP initiated the work and PP and RC designed the algorithms. RC developed the sub-starter generation and read coherence algorithms, while PP developed the other parts. RC and PP performed the experiments and wrote the paper. Both authors read and approved the final manuscript.

## Supplementary Material

Additional file 1 **Material and****Mapsembler****commands and results.** Click here for file
